# Co-inhibition of polo-like kinase 1 and Aurora kinases promotes mitotic catastrophe

**DOI:** 10.18632/oncotarget.3313

**Published:** 2015-03-20

**Authors:** Jingjing Li, Myung Jin Hong, Jeremy P.H. Chow, Wing Yu Man, Joyce P.Y. Mak, Hoi Tang Ma, Randy Y.C. Poon

**Affiliations:** ^1^ Division of Life Science, Center for Cancer Research, and State Key Laboratory of Molecular Neuroscience, Hong Kong University of Science and Technology, Clear Water Bay, Hong Kong

**Keywords:** anticancer drugs, antimitotic drugs, kinases, mitosis, mitotic slippage

## Abstract

Mitosis is choreographed by a number of protein kinases including polo-like kinases and Aurora kinases. As these kinases are frequently dysregulated in cancers, small-molecule inhibitors have been developed for targeted anticancer therapies. Given that PLK1 and Aurora kinases possess both unique functions as well as co-regulate multiple mitotic events, whether pharmacological inhibition of these kinases together can enhance mitotic catastrophe remains an outstanding issue to be determined. Using concentrations of inhibitors that did not induce severe mitotic defects on their own, we found that both the metaphase arrest and mitotic slippage induced by inhibitors targeting Aurora A and Aurora B (MK-5108 and Barasertib respectively) were enhanced by a PLK1 inhibitor (BI 2536). We found that PLK1 is overexpressed in cells from nasopharyngeal carcinoma, a highly invasive cancer with poor prognosis, in comparison to normal nasopharyngeal epithelial cells. Nasopharyngeal carcinoma cells were more sensitive to BI 2536 as a single agent and co-inhibition with Aurora kinases than normal cells. These observations underscore the mechanism and potential benefits of targeting PLK1 and Aurora kinases to induce mitotic catastrophe in cancer cells.

## INTRODUCTION

Accurate cell division relies on the actions of a well-balanced network of protein kinases and phosphatases [1]. Polo-like kinases and Aurora kinases are two of the most studied families of mitotic kinases. These kinases performs multiple functions in mitosis, including centrosome maturation, kinetochore-spindle attachment, chromosome segregation, and cytokinesis.

One of the critical functions of polo-like kinase 1(PLK1) in mitosis is for kick-starting the autocatalytic loop that controls the activity of cyclin B1–CDK1 [2]. Phosphorylation by PLK1 promotes the activation of CDC25 [3–5] and inactivation of WEE1 [6,7]. In addition to regulating cyclin B1–CDK1 activity through the CDC25/WEE1 axis, PLK1 also controls the localization of cyclin B1 by phosphorylating its nuclear export sequence. This inhibits the binding of the export receptor CRM1, thereby triggering nuclear accumulation and activation of cyclin B1 during prophase [8].

Similarly to many protein kinases, PLK1 activation requires phosphorylation of a residue on the T-loop (Thr210). PLK1^Thr210^ is phosphorylated by Aurora A (also called AURKA), an event that is assisted by Bora [9]. PLK1 in turn promotes the recruitment of AURKA to the centrosomes in late G_2_ [10]. PLK1 also phosphorylates Bora, generating a phosphodegron motif that is recognized by the ubiquitin ligase SCF^β–TrCP^, thereby triggering Bora destruction [11]. Degradation of Bora is believed to be important for redistributing AURKA from a cytoplasmic Bora-containing complex to a TPX2-containing complex at the mitotic spindle. The TPX2–AURKA complex then promotes centrosome maturation and bipolar spindle formation in a Ran- GTP-dependent manner [12].

Although highly related to AURKA, Aurora B (also called AURKB) is a component of the chromosomal passenger complex (CPC), which is comprised of AURKB, INCENP, borealin, and survivin [13]. CPC localizes to chromosomes and kinetochores in early mitosis and functions in chromosome–microtubule interactions, sister chromatid cohesion, and the spindle-assembly checkpoint. In early mitosis, PLK1^Thr210^ is phosphorylated by AURKB at centromeres and kinetochores [14]. In anaphase, the CPC is relocated to the mid-zone to promote cytokinesis.

The activity of AURKA increases from late G_2_ phase onwards and peaks during prometaphase. On the other hand, the activity of AURKB peaks from metaphase to the end of mitosis. Activation of AURKA requires binding to specific cofactors including Ajuba, Bora, and TPX2, leading to the autophosphorylation of a residue in the T-loop (Thr288) [15]. Similarly, AURKB is activated by autophosphorylation of the T-loop (Thr232) after binding to members of the CPC [16]. At the end of mitosis, both AURKA and AURKB are degraded by APC/C-mediated ubiquitination.

Although PLK1, AURKA, and AURKB have their unique functions during mitosis, they also co-regulate multiple processes, including entry into mitosis, mitotic spindle formation, sister chromatid resolution, chromosome–spindle connections, and cytokinesis. Mechanistically, these kinases often phosphorylate different components involve in the same mitotic process [17].

As Aurora kinases are upregulated in several human cancers and correlated with poor prognosis, they are believed to be important anticancer drug targets [18]. More than 20 small-molecule Aurora kinase inhibitors have been developed and are at various stages of clinical trials [19]. While the early generations of Aurora kinase inhibitors inactivate both AURKA and AURKB indiscriminately, later generations of inhibitors are able to target AURKA or AURKB specifically. Similarly, scores of anticancer drug candidates targeting PLK1 have been developed [20].

Inhibition of PLK1 or the two Aurora kinases triggers a process generally termed mitotic catastrophe. More recent attempts to standardize the term mitotic catastrophe defined it to be associated with aberrant mitotic activity that ultimately triggers cell death or irreversible cell cycle arrest [21]. In this definition, cell death can occur either during or after the defective mitosis such as mitotic slippage.

Given the close relationship between PLK1 and the Aurora kinases, a salient question is the biological consequences when these kinases are targeted together. Will inhibitors of PLK1 and Aurora kinases synergistically enhance a particular mitotic defect? Or will they antagonize each other, causing different defects as if they act independently? Here we address these questions directly at single cells level using live-cell imaging, in particular comparing normal and cancer cells from nasopharyngeal carcinoma.

## RESULTS

### The activity of PLK1 is impaired in the absence of Aurora kinases

**Figure 1:**
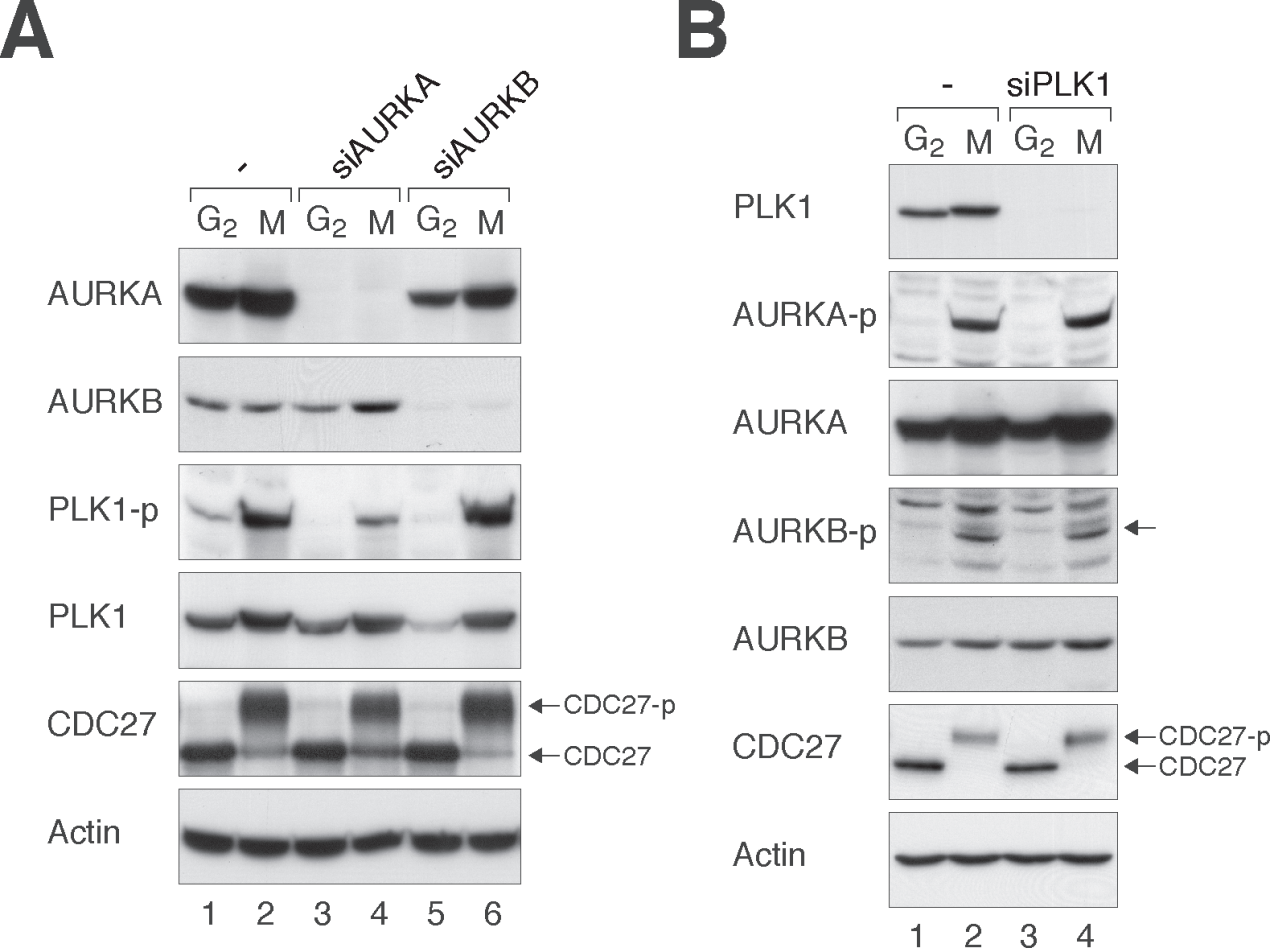
Loss of Aurora kinases disrupts PLK1 activity during mitosis **(A)** Depletion of AURKA impairs the activation of PLK1. HeLa cells were transfected with control siRNA, siAURKA, or siAURKB. The cells were enriched in G_2_ phase or mitosis as described in Materials and Methods. Lysates were prepared and the indicated proteins were detected with immunoblotting. CDC27 analysis was included as a marker of mitosis. The positions of the unphosphorylated and mitotic form of CDC27 are indicated. Uniform loading of lysates was confirmed by immunoblotting for actin. **(B)** Depletion of PLK1 does not affect the activation of AURKA or AURKB. HeLa cells were transfected with control siRNA or siPLK1, before enriched in G_2_ phase or mitosis as described in Materials and Methods. Lysates were prepared and the indicated proteins were detected with immunoblotting.

To determine the relationship between Aurora kinases and PLK1 during G_2_ phase and mitosis, we first downregulated the Aurora kinases using specific siRNAs. After HeLa cells were transfected with siRNAs against AURKA and AURKB (siAURKA and siAURKB respectively), they were synchronized at either G_2_ phase (using a double thymidine block–release procedure) or mitosis (using a procedure involving nocodazole followed by mechanical shake-off). Lysates were prepared and the activity of PLK1 was determined through the level of PLK1^Thr210^ phosphorylation. Figure 1A shows that depletion of AURKA reduced PLK1^Thr210^ phosphorylation without affecting the abundance of total PLK1, confirming that the presence of AURKA was important for PLK1 activation during mitosis. In contrast, depletion of AURKB did not significantly affect PLK1 activity. The immunoblotting analysis also confirmed the efficient depletion of AURKA and AURKB by the siRNAs.

We also performed the converse experiment by analyzing the effects of PLK1 depletion on the activation of Aurora kinases. Figure 1B shows that PLK1 was effectively depleted with siPLK1 in both G_2_ and mitosis. However, the loss of PLK1 affected neither the total nor the activated forms of Aurora kinases (AURKA^Thr288^ and AURKB^Thr232^) during mitosis.

These results highlighted the link between AURKA and PLK1. Although AURKB did not affect PLK1 activation, it is possible that the two proteins may act on common targets. These observations suggested the possibility of synergism if Aurora kinases and polo-like kinases are targeted together.

### Pharmacological inhibition of PLK1 with BI 2536 induces metaphase defects and mitotic catastrophe

**Figure 2:**
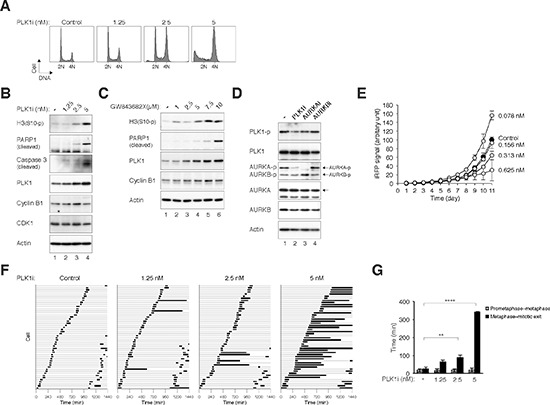
Inhibition of PLK1 with BI 2536 induces metaphase defects and mitotic catastrophe **(A)** Inhibition of PLK1 promotes G_2_/M delay and apoptosis in a dose-dependent manner. HeLa cells were incubated with buffer or the indicated concentrations of BI 2536 (PLK1i). After 24 h, the cells were harvested and the DNA content was analyzed with flow cytometry. The positions of 2N and 4N DNA content are indicated. **(B)** Inhibition of PLK1 induces mitotic catastrophe. HeLa cells were incubated with buffer or the indicated concentrations of PLK1i for 24 h. Lysates were prepared and the indicated proteins were detected with immunoblotting. Actin analysis was included to assess protein loading and transfer. **(C)** Treatment with the PLK1 inhibitor GW843682X induces mitotic catastrophe. HeLa cells were incubated with buffer or the indicated concentrations of GW843682X for 24 h. Lysates were prepared and the indicated proteins were detected with immunoblotting. **(D)** Inhibition of PLK1 affects AURKA activity. Mitotic HeLa cells were isolated by treating cells with nocodazole for 16 h followed by mechanical shake off. The cells were either untreated or incubated with PLK1i, AURKAi, or AURKBi. The proteasome inhibitor MG132 was added to prevent the cells from exiting mitosis. The cells were harvested after 2 h. Lysates were prepared and the indicated proteins were detected with immunoblotting. **(E)** Low concentrations of PLK1i stimulate cell proliferation. HeLa cells expressing iRFP (~200 cells) were seeded onto 6-well culture plates and cultured in the presence of buffer or different concentrations of PLK1i. On different days, the plate was scanned with an Odyssey infrared imaging system and the iRFP signal was quantified (average± SD of three independent experiments). Note that the PLK1i was left in the medium continuously throughout the experiment. At 0.078 nM, PLK1i significantly increased cell proliferation (*P* < 0.001; Student's *t*-test). **(F)** Inhibition of PLK1 induces a delay in mitosis. HeLa cells expressing histone H2B-GFP were exposed to buffer or the indicated concentrations of PLK1i. Individual cells were then tracked for 24 h with time-lapse microscopy. Each horizontal bar represents one cell (*n* = 50). Light grey: interphase; black: mitosis (from DNA condensation to anaphase or mitotic slippage); truncated bars: cell death. **(G)** PLK1i inhibits metaphase–anaphase transition. Cells were treated and imaged as described in panel (F). The duration from prometaphase to metaphase and from metaphase to the end of mitosis (anaphase, apoptosis, or the end of imaging period) was quantified (average ±90% CI). PLK1i treatment significantly extended mitosis after the metaphase was formed (****: *P* < 0.0001; **: *P* < 0.01; Student's *t-*test).

To study possible synergism between the antimitotic effects of inhibitors of PLK1 and Aurora kinases, newer generation of small-molecule inhibitors that show relatively high specificity were used in this study. We first verified the effects of an inhibitor of PLK1 called BI 2536 [22,23] (designated PLK1i herein) as a single agent on the cell cycle. Flow cytometry analysis revealed that PLK1i induced a G_2_/M cell cycle delay in a dose-dependent manner (Figure 2A). Mitotic delay was confirmed by the increase in histone H3^Ser10^ phosphorylation (Figure 2B). Moreover, the stimulation of apoptosis (as indicated by PARP1 cleavage, caspase 3 cleavage, and increase of sub-G_1_ population) suggested that mitotic catastrophe was induced by PLK1i in these cells (Figure 2B). PLK1 itself accumulated upon incubation with PLK1i, possibly reflecting the increase of mitotic population (see below). Similar induction of accumulation of PLK1, histone H3^Ser10^ phosphorylation, and PARP1 cleavage were obtained by targeting PLK1 with another inhibitor called GW843682X [24], indicating that the effects were not specific for BI 2536 only (Figure 2C). Another PLK1 inhibitor called BI 6727 (also called Volasertib) [25] also induced mitotic block and apoptosis in a dose-dependent manner (Supplementary Figure S1).

We found that treatment with PLK1i reduced the activation of AURKA during mitosis (as indicated by AURKA^Thr288^ phosphorylation) (Figure 2D), further suggesting the link between PLK1 and AURKA. This effect on AURKA differed from the results obtained using siRNA-mediated downregulation of PLK1 (Figure 1B). One possibility is that siRNA-inhibition of PLK1 was not as complete as PLK1i. Alternatively, other PLK1i targets such as other members of the polo-like kinase family may also be involved in AURKA activation.

To analyze the effects of PLK1i on cell growth, we used a recently developed method based on an infrared fluorescent protein reporter [26]. Infrared-based detection has several advantages over conventional proliferation assays, including higher sensitivity, allowing time-dependent measurement, and lower stress to the cells. As expected, PLK1i reduced cell proliferation in a dose-dependent manner above 0.156 nM (Figure 2E). Interestingly, cell proliferation was in fact stimulated in the presence of lower concentrations of PLK1i. Cell proliferation was similarly stimulated with a low concentration of PLK1i in another cell line (HONE1), indicating that the effect was not limited to HeLa cells (Supplementary Figure S2).

To evaluate precisely how mitosis was disrupted by PLK1i, the fate of individual cells was followed using live-cell imaging. Both cell and DNA morphology were analyzed. Figure 2F shows that compare to control cells, the duration of mitosis was increased in the presence of increasing concentration of PLK1i. Mitosis was extended predominantly at the metaphase–anaphase transition (Figure 2G). Examples of control cells (Supplementary Video 1) and PLK1i-treated cells (Supplementary Video 2) are shown in the Supplemental data. PLK1i-treated cells typically remained in metaphase for a long period of time (on average >300 min, ten times longer than the duration of normal mitosis) before undergoing apoptosis. The impression of periodic loss of chromosomal alignment in the time-lapse videos was due to the metaphase plate flipping on its axis during the arrest).

Several early studies of PLK1 inhibitors indicated that inhibition of PLK1 induced a cell cycle arrest at early mitosis instead of at metaphase. For example, BI 2536 was found to induce a prometaphase with aberrant monopolar spindles followed by mitotic catastrophe [23]. To address this discrepancy with our results, we also tested if higher concentrations of PLK1i affected other aspects of mitosis. Supplementary Figure S3 shows that 100 nM of PLK1i induced a complete mitotic arrest followed by cell death. At this concentration of PLK1i, metaphase plate was unable to form and the cells were arrested in a prometaphase-like state before undergoing apoptosis (Supplementary Video 3). At even higher concentrations of PLK1i (1 μM), the cells underwent apoptosis without entering mitosis (data not shown).

Collectively, these data indicate that inhibition of PLK1 with BI 2536 at low concentrations induces a profound delay in metaphase–anaphase transition followed by apoptosis.

### Cooperation between inhibitors of PLK1 and Aurora kinases in mitotic catastrophe

Given the evidence of potential links between PLK1 and the Aurora kinases, we next determined if there is synergism between PLK1i and Aurora kinase inhibitors. In this study, newer generation of Aurora kinase inhibitors that could distinguish different members of the family were used. By detecting the activated forms of AURKA/AURKB, we recently demonstrated that MK-5108 (also called VX-689) inhibits AURKA without affecting AURKB [27]. On the other hand, Barasertib (also called AZD1152-HQPA) specifically inactivated AURKB without affecting AURKA over a wide range of concentrations [27].

**Figure 3:**
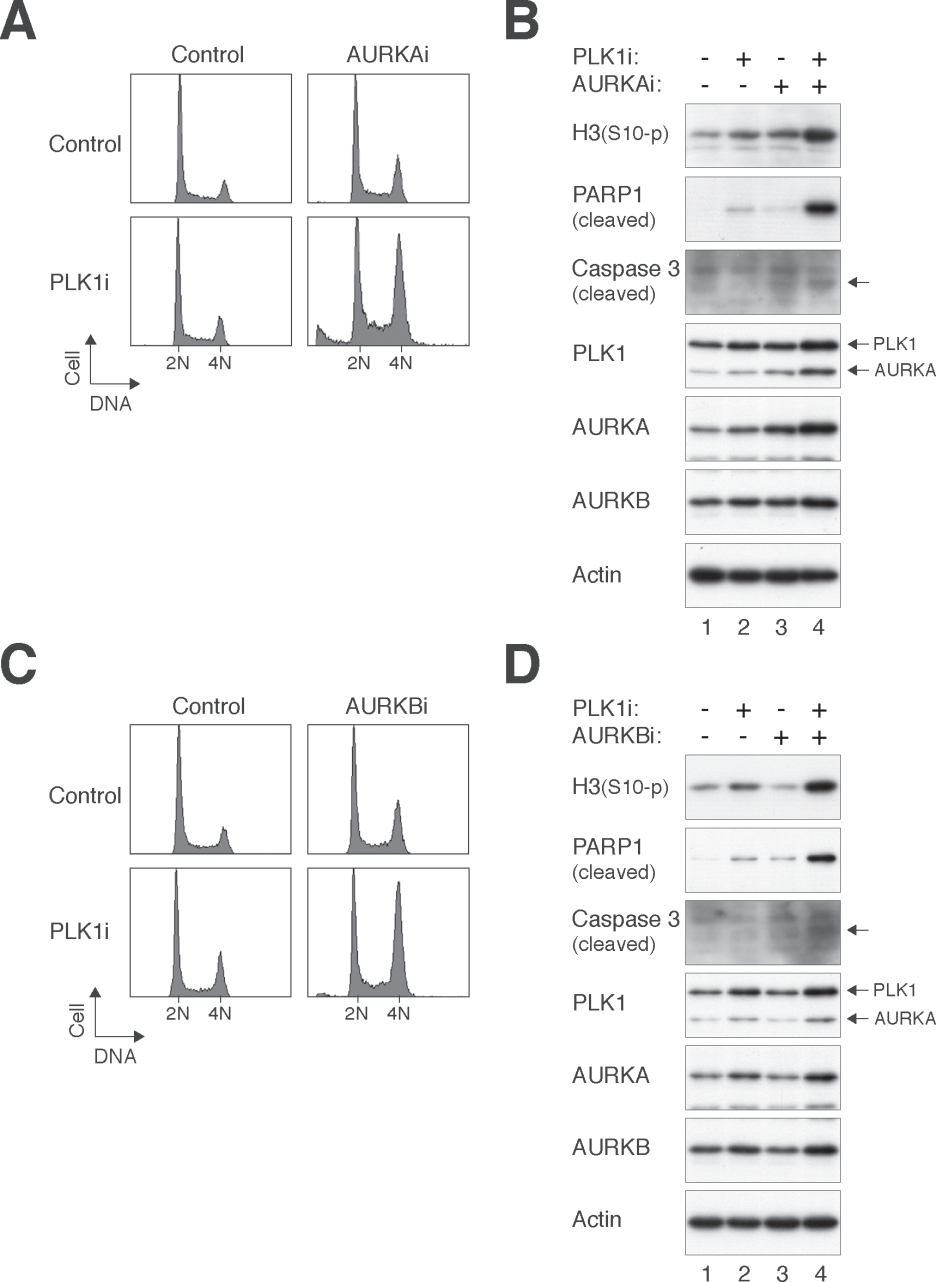
PLK1i cooperates with Aurora kinase inhibitors to induce mitotic catastrophe **(A)** PLK1i cooperates with AURKAi to induce G_2_/M delay. HeLa cells were incubated with PLK1i (2.5 nM) and/or AURKAi (250 nM) as indicated. After 24 h, the cells were harvested and analyzed with flow cytometry. The positions of 2N and 4N DNA content are indicated. **(B)** Mitotic catastrophe induced by PLK1i and AURKAi. Cells were treated as described in panel (A). Lysates were prepared and the indicated proteins were detected with immunoblotting. Note that the PLK1 blot was performed after probing the membrane with AURKA antibodies. Equal loading of lysates was confirmed by immunoblotting for actin. **(C)** PLK1i cooperates with AURKBi to induce G_2_/M delay. HeLa cells were incubated with PLK1i (2.5 nM) and/or AURKBi (12.5 nM) as indicated. After 24 h, the cells were harvested and analyzed with flow cytometry. **(D)** Mitotic catastrophe induced by PLK1i and AURKBi. Cells were treated as described in panel (C). Lysates were prepared and the indicated proteins were detected with immunoblotting. Note that the PLK1 blot was performed after probing the membrane with AURKA antibodies (the positions of PLK1 and AURKA are indicated). Equal loading of lysates was confirmed by immunoblotting for actin.

Relative low concentrations of the PLK1i and Aurora kinase inhibitors were used with the aim of not inducing mitotic defects on their own. Figure 3A shows that while the cell cycle profiles of cells treated with PLK1i or MK-5108 (AURKAi herein) individually were similar to control cells, a significant G_2_/M delay was induced after the two chemicals were added together. Protein analysis verified that at these relatively low concentrations, PLK1i and AURKAi individually only increased histone H3^Ser10^ phosphorylation marginally (Figure 3B). By contrast, histone H3^Ser10^ phosphorylation and cleavage of PAPR1 and caspase 3 were increased in the presence of both PLK1i and AURKAi, suggesting that the two drugs acted cooperatively to induce mitotic catastrophe. Similarly, Barasertib (AURKBi herein) acted cooperatively with PLK1i to induce accumulation of G_2_/M population (Figure 3C), phosphorylated histone H3^Ser10^, and cleaved PARP1 (Figure 3D).

Similar cooperation was found between another PLK1 inhibitor (BI 6727) and AURKAi (Supplementary Figure S4A) and AURKBi (Supplementary Figure S4B) in inducing G_2_/M delay, indicating that the effect was not restricted to PLK1i.

**Figure 4:**
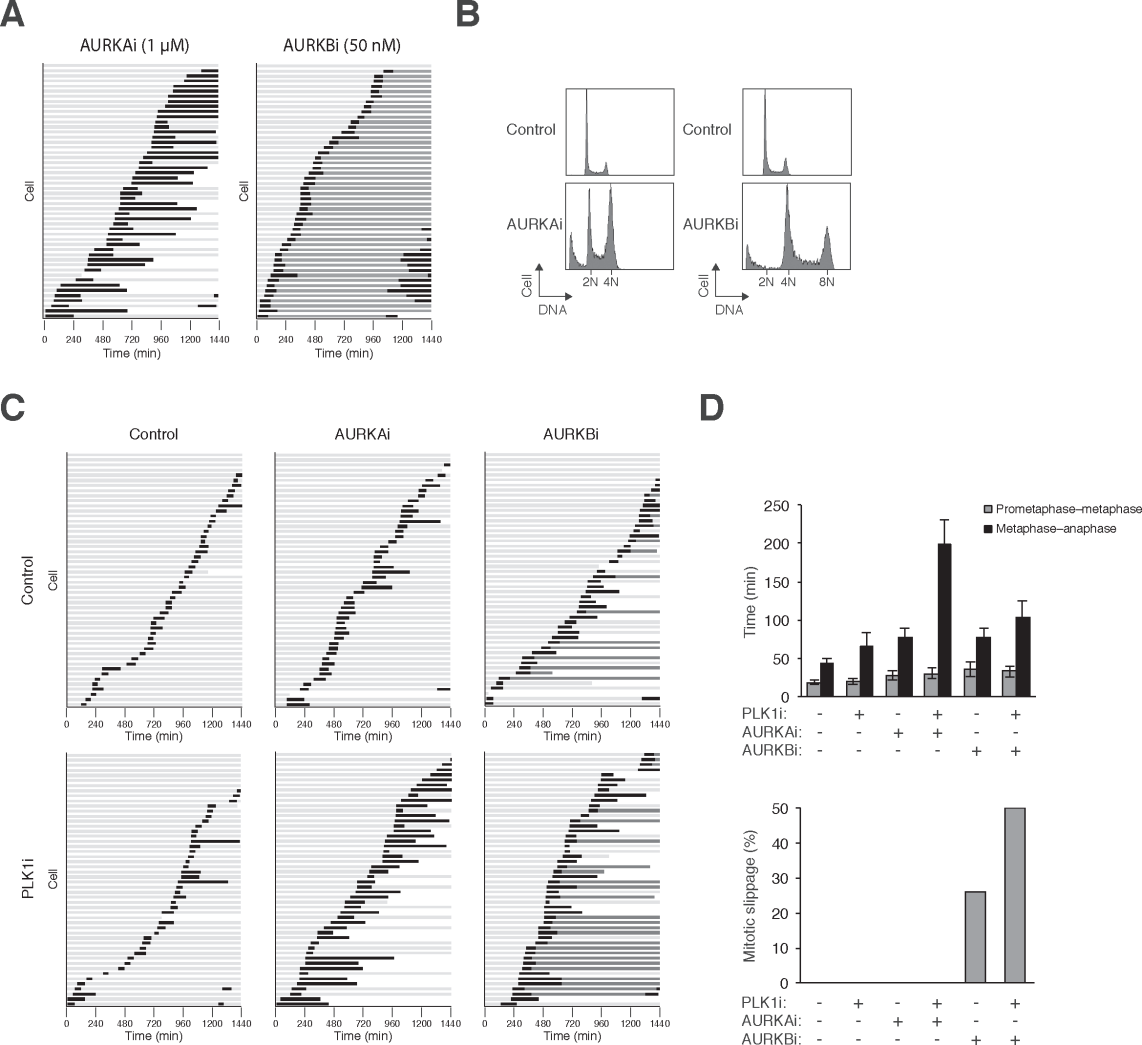
PLK1i cooperates with Aurora kinase inhibitors to induce mitotic arrest and slippage **(A)** Inhibition of AURKA and AURKB triggered mitotic arrest and mitotic slippage respectively. HeLa cells expressing histone H2B-GFP were incubated with AURKAi (1 μM) or AURKBi (50 nM). Individual cells were then tracked for 24 h with time-lapse microscopy. Each horizontal bar represents one cell (*n* = 50). Light grey: interphase; black: mitosis (from DNA condensation to anaphase or mitotic slippage); dark grey: mitotic slippage; truncated bars: cell death. **(B)** Cells were treated with AURKAi or AURKBi as described in panel (A). After 24 h, the cells were harvested and analyzed with flow cytometry. The positions of 2N, 4N, and 8N DNA content are indicated. **(C)** PLK1i cooperates with Aurora kinase inhibitors to induce mitotic arrest and slippage. HeLa cells expressing histone H2B-GFP were incubated with PLK1i (2.5 nM), AURKAi (250 nM), and AURKBi (12.5 nM). Individual cells were then tracked for 24 h with time-lapse microscopy. Each horizontal bar represents one cell (*n* = 50). Light grey: interphase; black: mitosis (from DNA condensation to anaphase or mitotic slippage); dark grey: mitotic slippage; truncated bars: cell death. **(D)** Cells were treated and imaged as described in panel (C). The duration of mitosis (from prometaphase–metaphase and from metaphase–anaphase was quantified (average ±90% CI; *n* = 50). The percentage of cells that underwent mitotic slippage was also quantified (lower panel).

To understand how the co-inhibition of PLK1 and Aurora kinases affected mitosis at single cells level, live-cell imaging was used to track individual cells. As a preamble, we first analyzed mitosis when the kinases were completely inhibited using relatively high concentrations of the drugs. As described above, inhibition of PLK1 induced a metaphase arrest (Figure 2; Supplementary Video 3). Inhibition of AURKA causes defects in centrosome separation and spindle formation, resulting in mitotic arrest and apoptosis [28]. Both live-cell imaging (Figure 4A) and flow cytometry (Figure 4B) supported that inhibition of AURKA induced mitotic arrest after metaphase plate formation (Supplementary Video 4). Co-inhibition of PLK1 and AURKA (using lower concentrations of the drugs) also delayed mitosis after the metaphase plate was formed (Figure 4D; Supplementary Video 5). In agreement with this, conventional immunostaining analysis indicated that the percentage of mitotic cells containing bipolar spindle remained unchanged after co-inhibition of PLK1 and AURKA (data not shown).

Due to the different functions of AURKA and AURKB, the effects of their pharmacological inactivation are very different. Inhibition of AURKB interferes with histone H3 phosphorylation, chromosome segregation, and cytokinesis, causing the formation of polyploid cells [28]. Accordingly, AURKBi triggered a process termed mitotic slippage, in which DNA decondensation occurred in the absence of sister chromatid separation (Figure 4A; see Supplementary Video 6). As a consequent of mitotic slippage, DNA rereplication occurred following AURKB inhibition (Figure 4B). Live-cell imaging further validated that mitotic slippage occurred after the metaphase plate formation (see Figure 4D).

Given that PLK1i and AURKBi affected different aspects of mitosis, we also investigated the effects on mitosis when the two chemicals were added together. Figure 4C shows that mitotic slippage was enhanced when both PLK1 and AURKB were co-inhibited (quantified in Figure 4D), suggesting that the effects of combinatorial treatment mostly reassembled that of AURKBi. An example of cells undergoing mitotic slippage following incubation with AURKBi and PLK1i is shown in Supplementary Video 7.

Taken together, PLK1i promoted the metaphase arrest and mitotic slippage induced by AURKAi and AURKBi, respectively.

### Targeting PLK1 and Aurora kinases specifically sensitizes nasopharyngeal carcinoma cells over normal epithelial cells

**Figure 5:**
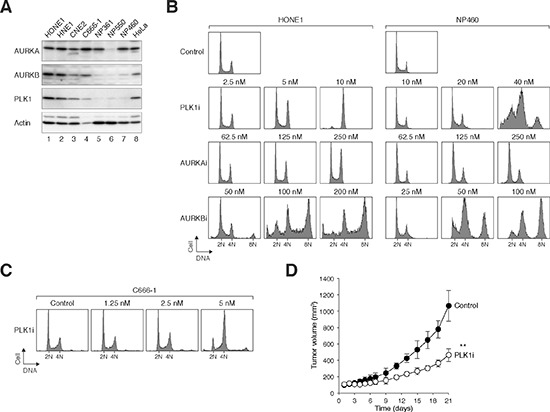
NPC cells are more sensitive to PLK1i than normal NP epithelial cells **(A)** PLK1 and AURKB are overexpressed in nasopharyngeal carcinoma (NPC) cell lines. Several NPC (HONE1, HNE1, CNE2, and C666-1) and immortalized normal nasopharyngeal (NP) cell lines (NP361, NP550, and NP460) were analyzed. Lysates from HeLa cells were also loaded for comparison. Cell-free extracts were prepared and the indicated proteins were detected by immunoblotting. **(B)** HONE1 cells are more sensitive than normal NP cells to PLK1i. HONE1 and normal NP NP460 cells were incubated with various concentrations of PLK1i, AURKAi, or AURKBi. After 24 h (for HONE1) or 48 h (for NP460), the cells were harvested and analyzed with flow cytometry. Control cells are shown on the top. Note that different concentrations of the drugs were used for the two cell lines to illustrate that HONE1 was more sensitive to PLK1i than NP460. **(C)** C666-1 cells are sensitive to PLK1 inhibition. C666-1 cells were incubated with the indicated concentrations of PLK1i. After 48 h, the cells were harvested and analyzed with flow cytometry. **(D)** PLK1i inhibits tumor growth in mouse xenograft models. HONE1 cells were injected subcutaneously into nude mice. PLK1i was delivered at the indicated time points as described in Materials and Methods. The volume of the tumor was measured on different days (control, black circle, *n* = 6; PLK1i, white circle, *n* = 6) (mean±SD). Treatment with PLK1i significantly reduced tumor growth (**: *P* < 0.01; paired *t*-test).

Given that targeting PLK1 and Aurora kinases resulted in cytotoxicity in cancer cells (HeLa), we next evaluated the cytotoxicity on a cancer *versus* normal cells model. Nasopharyngeal carcinoma (NPC) is a highly invasive cancer with poor prognosis. Although NPC is relatively rare in most parts of the world, high incidence rates are found in southern China and Southeast Asia. Many components of the cell cycle including the DNA damage checkpoint are altered in NPC [29]. To study if PLK1, AURKA, and AURKB are dysregulated in NPC cells, lysates from different NPC cell lines (C666-1, CNE2, HNE1, and HONE1) were prepared and analyzed with immunoblotting using specific antibodies (Figure 5A). Several lines of normal nasopharyngeal (NP) epithelial cells immortalized with telomerase (NP361, NP460, and NP550) were used as a comparison. Both PLK1 and AURKB were found to be overexpressed in the NPC cell lines. On the other hand, the expression of AURKA was similar in NPC and normal cell lines (apart from a low expression in NP550).

We first analyzed if NPC and normal NP cells have similar sensitivity to inhibitors of PLK1 and Aurora kinases when they were used alone. NPC (HONE1) or normal NP cells (NP460) were treated with different concentrations of the inhibitors and analyzed with flow cytometry (Figure 5B). We found that HONE1 cells were more sensitive to PLK1i than NP460: while 10 nM was sufficient to induce a mitotic defect in HONE1 cells, 40 nM was required for NP460. By contrast, HONE1 and NP460 showed comparable sensitivity to AURKAi and AURKBi. The higher sensitivity of NPC cells to PLK1i was also confirmed with another NPC cell line (C666-1, an Epstein-Barr virus-positive NPC cell line) (Figure 5C).

Given the sensitivity of NPC cells to PLK1i, we further tested the growth inhibition of PLK1i on NPC cells with nude mouse xenograft models. HONE1 cells were injected subcutaneously into nude mice; and PLK1i was delivered using a fractionated dose approach. Figure 5D shows that treatment with PLK1i reduced the rate of tumor growth, indicating that PLK1i exerted a strong tumor-inhibitory activity in NPC xenograft mouse models.

**Figure 6:**
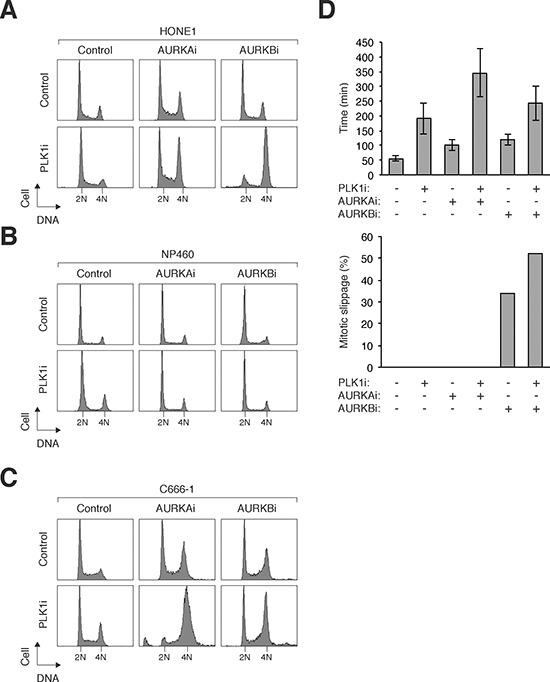
Co-inhibition of PLK1 and Aurora kinases specifically sensitizes NPC cells **(A)** Co-inhibition of PLK1 and Aurora kinases induces mitotic defects in NPC cells. HONE1 cells were incubated with a combination of PLK1i (2.5 nM), AURKAi (250 nM), and AURKBi (25 nM). After 24 h, the cells were harvested and analyzed with flow cytometry. **(B)** NP460 cells are not affected by combinatorial treatment with PLK1i, AURKAi, and AURKBi. NP460 cells were incubated with a combination of PLK1i (2.5 nM), AURKAi (250 nM), and AURKBi (25 nM). After 24 h, the cells were harvested and analyzed with flow cytometry. **(C)** C666-1 cells are sensitive to co-inhibition of PLK1 and Aurora kinases. C666-1 cells were incubated with a combination of PLK1i (2.5 nM), AURKAi (500 nM), and AURKBi (20 nM). After 48 h, the cells were harvested and analyzed with flow cytometry. **(D)** PLK1i cooperates with Aurora kinase inhibitors to induce mitotic arrest and slippage. HONE1 cells expressing histone H2B-mRFP were incubated with PLK1i (2.5 nM), AURKAi (250 nM), and AURKBi (25 nM). Individual cells were then tracked for 24 h with time-lapse microscopy (the fate of individual cells was shown in Supplementary Figure S5A). The duration of mitosis (from prometaphase–anaphase) was quantified (average ±90% CI; *n* = 50). The percentage of cells that underwent mitotic slippage was also quantified (lower panel).

To determine if NPC cells could be sensitized by co-inhibition of PLK1 and Aurora kinases, the respective inhibitors were used at relative low concentrations that did not elicit a significant response on their own. Similar to HeLa cells (Figure 3A), challenging HONE1 cells with PLK1i together with AURKAi or AURKBi induced mitotic defects (Figure 6A). In marked contrast, the same concentrations of AURKAi or AURKBi added together with PLK1i did not affect the normal NP460 cells (Figure 6B). Inhibition of PLK1 and AURKA/AURKB also resulted in defective mitosis in another NPC cell line (C666-1) (Figure 6C), excluding the possibility that the effect was only specific to HONE1 cells.

Enhancement of the effects of AURKAi and AURKBi by co-inhibition of PLK1 was further confirmed using live-cell imaging. PLK1i increased the mitotic delay induced by AURKAi and the mitotic slippage induced by AURKBi (Figure 6D; the fate of individual cells are shown in Supplementary Figure S5A). In contrast, the same concentrations of AURKAi or AURKBi added together with PLK1i did not affect NP460 cells (Supplementary Figure S5B).

**Figure 7:**
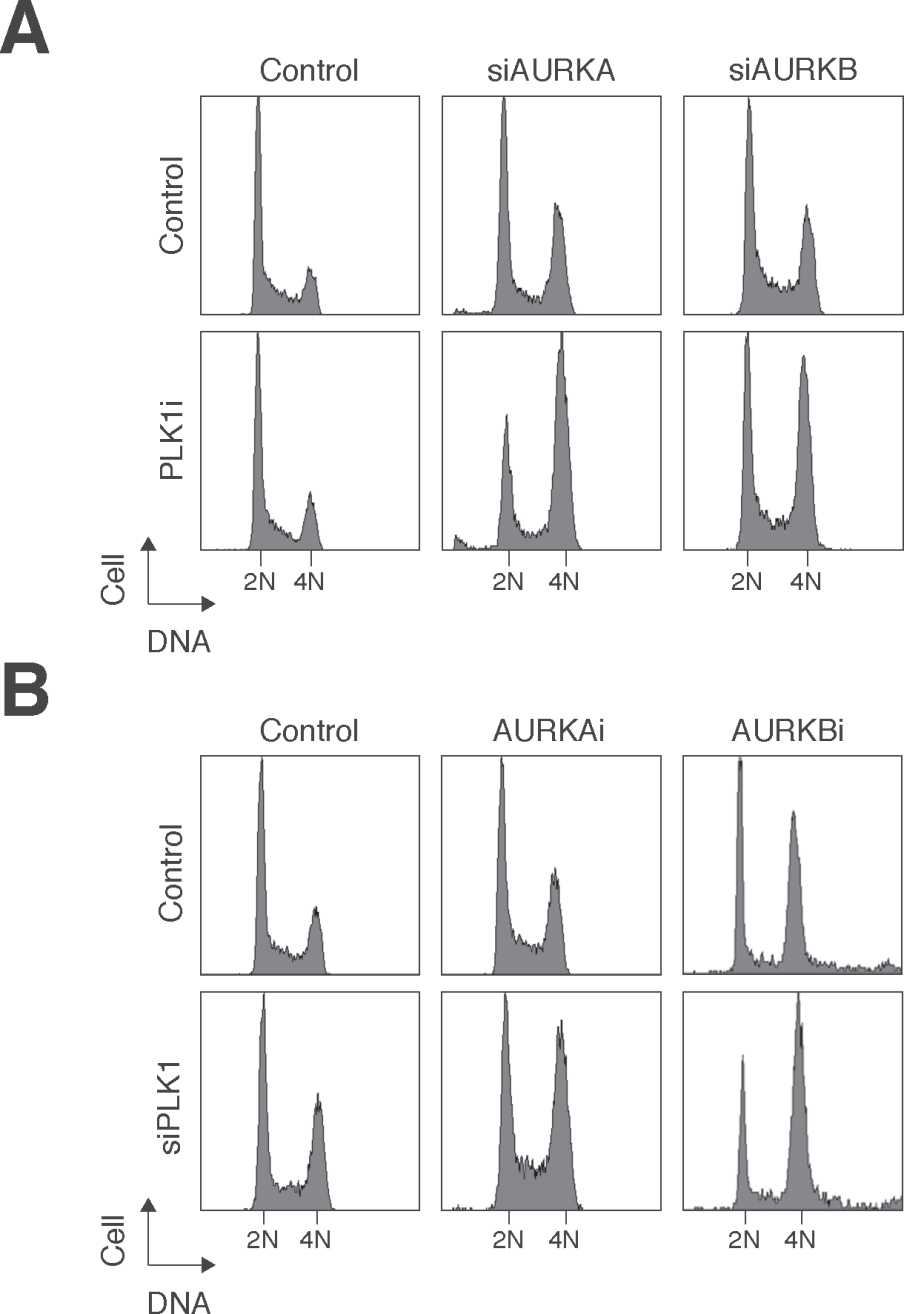
Small-molecule inhibitors and siRNAs of PLK1 and Aurora kinases act synergistically **(A)** Depletion of Aurora kinases increased the sensitivity to PLK1i. HONE1 cells were transfected with control siRNA, siAURKA, or siAURKB. After 24 h, the cells were treated with either buffer or PLK1i (2.5 nM). After 24 h, the cells were harvested and analyzed with flow cytometry. **(B)** Depletion of PLK1 increased the sensitivity to Aurora kinase inhibitors. HONE1 cells were transfected with either control siRNA or siPLK1. After 24 h, the cells were treated with buffer, AURKAi (500 nM), or AURKBi (10 nM). After 24 h, the cells were harvested and analyzed with flow cytometry.

To determine if the effects of the PLK1 and Aurora kinase inhibitors are specific for the respective kinases, we replaced one of the inhibitors with siRNA against the kinase (see Figure 1 for the efficacy of the siRNAs). The challenge of this experiment was that depletion of PLK1 and AURKA with siRNAs already triggered extensive mitotic defects. Hence relatively low concentrations of the siRNAs were used in these experiments. Figure 7A shows that the presence of PLK1i enhanced the mitotic defects caused by downregulation of AURKA or AURKB. Conversely, downregulation of PLK1 with siRNA increased the mitotic defects caused by AURKAi and AURKBi (Figure 7B).

Collectively, these data show that PLK1 is overexpressed in NPC cells. NPC cells are also more sensitive to PLK1i as a single agent than normal NP cells. Accordingly, NPC cells are also significantly more sensitive to co-inhibition of PLK1 and Aurora kinases than normal cells.

## DISCUSSION

In this study, we provided several lines of evidence suggesting that combining inhibitors of PLK1 and Aurora kinases induced more mitotic defects than individual inhibitors alone. This was illustrated by an increase in G_2_/M population in multiple cell lines including HeLa (Figure 3A, 3C), HONE1 (Figure 6A), and C666-1 (Figure 6C). As cells in G_2_ phase, mitosis, or after mitotic slippage all contained the same amount of DNA, time-lapse microscopy was used to further distinguish the different mitotic defects. These analyses indicated that PLK1i induced different effects when combined together with either AURKAi or AURKBi: while PLK1i and AURKAi together delayed mitotic exit, PLK1i and AURKBi promoted mitotic slippage (Figure 4C, 4D). These results suggest the possibility of synergism when PLK1 and either AURKA or AURKB are targeted together.

The molecular basis of synergism between inhibitors of PLK1 and Aurora kinases is likely to be multifaceted. It can be considered both at the level of the kinases themselves and the pathways they regulated. Progress in the past several years has unraveled some of the underlying principles of mutual regulation between PLK1 and Aurora kinases. With the help of Bora, the activating residue on the T-loop of PLK1 (Thr210) can be phosphorylated directly by AURKA during late G_2_ [9,30]. This probably occurs at the centrosomes, where both AURKA and PLK1 are localized. Another protein called Furry (FRY) also binds PLK1 and AURKA, facilitating the AURKA-mediated phosphorylation of PLK1^Thr210^ [31]. In early mitosis, PLK1 at centromeres and kinetochores is activated by AURKB [14]. This is crucial for PLK1 function in regulating chromosome dynamics in prometaphase. Given both AURKA and AURKB can activate PLK1, it is not unexpected that small-molecule inhibitors PLK1 and AURKA or AURKB can cooperate in suppressing PLK1 functions.

Conversely, inhibition of PLK1 could affect the activities of AURKA and AURKB. The activating T-loops of AURKA and AURKB, however, are not directly phosphorylated by PLK1 (they are carried out by autophosphorylation). While PLK1 may not affect AURKA and AURKB kinase activity directly, it may regulate their functions by other mechanisms. For example, PLK1 promotes the recruitment of AURKA to the centrosomes in late G_2_ [10]. Phosphorylation of Bora by PLK1 initiates a pathway leading to the redistribution of AURKA from a cytoplasmic Bora-containing complex to a TPX2-containing complex that is important for centrosome maturation and bipolar spindle formation [11]. In *Xenopus*, phosphorylation of TPX2 by PLK1 also enhances the activation of AURKA [32]. Proteomic analysis of substrates confirmed that PLK1 inactivation indeed results in a reduction of AURKA activity [33].

Likewise, PLK1 can also regulate AURKB indirectly by phosphorylating other proteins. For example, phosphorylation of survivin by PLK1 is involved in the activation of AURKB [34]. PLK1 can also regulate the abundance of AURKB by phosphorylating FoxM1; this increases the transcriptional activity of FoxM1, which is required for the expression of key mitotic regulators including AURKB [35].

In addition of their mutual regulation, an emerging view is that PLK1 cooperates with Aurora kinases to regulate multiple stages of mitosis, including cohesion, centrosome maturation, and kinetochore–microtubule interaction [17]. The synergism displayed by targeting PLK1 and Aurora kinases together could possibly be due to a reduction of phosphorylation of different substrates in these mitotic events. Alternatively, individual proteins could be phosphorylated by both PLK1 and Aurora kinases (on different sites). For example, a phosphoproteome study identified several substrates common to both PLK1 and AURKA, including pericentrin, the γ-tubulin subunit GCP2, and the centrosomal protein Cep215 [33].

In this study, we used NPC as a model to investigate the specific sensitivity of cancer cells to targeting PLK1 and Aurora kinases. A rationale of using NPC as a cancer model is the availability of both cancerous and relatively normal immortalized epithelial cell lines for direct comparison, which are not commonly available for many cancer models. Another reason for studying this relatively rare cancer is the notable lack of effective chemotherapeutic treatment. We found that NPC cells (both C666-1 and HONE1) are more sensitive to PLK1i than normal nasopharyngeal epithelial cells (Figure 5B, 5C). Although this has important implication for anticancer therapies at least for this type of cancer, the underlying mechanism is not immediately obvious. One possibility is hinted by the expression of PLK1 in NPC cell lines. Compare to several normal nasopharyngeal epithelial cell lines, NPC cell lines contained overexpressed levels of PLK1 (Figure 5A). The elevated level of PLK1 in cancer cells could reflect a larger number of targets for PLK1i per cell. It could also reflect a stronger reliance of cancer cells on PLK1 for survival than in normal cells. This hypothesis can be tested by generating normal nasopharyngeal epithelial cells that ectopically express PLK1. However, overexpression of drug targets does not necessary result in higher drug sensitivity. For example, NPC cells contained higher level of AURKB than normal cells (Figure 5A), but both cell types were equally sensitive to AURKBi (Figure 5B).

Irrespective of the mechanism, the higher sensitivity of NPC cells to PLK1i offers a rationale for targeting PLK1 in the management of this cancer. In support of this, PLK1i could effectively reduce the growth of HONE1 cells in xenograft mouse models (Figure 5D). Given that PLK1i was already more effective in NPC cells than normal cells, it was even more effective in triggering mitotic defects when combined with AURKAi or AURKBi (Figure 6). We did not observe further synergism between PLK1i and AURKAi or AURKBi in the nude mice model because PLK1i alone already exerted strong tumor-suppressing activity (data not shown).

In conclusion, cancer cells appear to be more sensitive to pharmacological inhibition of PLK1 than normal cells. Mitotic catastrophe is further enhanced by co-inhibition of the Aurora kinases. These findings provide a foundation to support clinical studies of targeting PLK1 and Aurora kinase families together in anticancer therapies.

## MATERIALS AND METHODS

### Cell culture

The HeLa used in this study was a clone that expressed the tTA tetracycline repressor chimera [36]. Nasopharyngeal carcinoma cell lines C666-1 [37], CNE2 [38], HNE1 [39], and HONE1 [39] were obtained from NPC AoE Cell Line Repository (The University of Hong Kong). Cells were propagated in RPMI1640 (for C666-1) or Dulbecco's modified Eagle's medium (DMEM) (for other cell lines) supplemented with 10% (v/v) calf serum (Life Technologies, Carlsbad, CA, USA) (for HeLa), 5% (v/v) calf serum and 5% (v/v) fetal bovine serum (Life Technologies) (for CNE2 and HNE1), or 10% (v/v) fetal bovine serum (for other cell lines) and 50 U/ml penicillin-streptomycin (Life Technologies). Telomerase-immortalized nasopharyngeal epithelial cell lines NP361, NP460, and NP550 [40] were propagated in keratinocyte serum-free medium supplemented (Life Technologies) with 50% v/v Epilife (Sigma-Aldrich, St Louis, MO, USA). HeLa cells stably expressing histone H2B-GFP were generated as described previously [41]. HONE1 cells stably expressing iRFP were generated as described previously [42]. HONE1 expressing histone H2B-mRFP were generated as previously described [43]. A NP460 cell line expressing histone H2B-GFP was generated by infecting NP460 cells with histone H2B-GFP-expressing retroviruses in the presence of 5 μg/ml of polybrene (Sigma-Aldrich), followed by sorting using a flow cytometer (FACSAria II, Becton Dickinson, Franklin Lakes, NJ, USA).

Unless stated specifically, cells were treated with the following reagents from the indicated suppliers and at the indicated final concentration: Barasertib (Selleck Chemicals, Houston, TX, USA), BI 2536 (Selleck Chemicals), BI 6727 (Selleck Chemicals), GW843682X (Sigma-Aldrich) (10 μM), MG132 (Sigma-Aldrich) (10 μM), MK-5108 (Selleck Chemicals) (1 μM), nocodazole (Sigma-Aldrich) (0.1 μg/ml), and thymidine (2 mM). Cell-free extracts were prepared as described previously [44].

Synchronization at G_2_ or mitosis after transfection with siRNAs was performed as described [45]. Briefly, HeLa cells were synchronized at early S phase with a double thymidine procedure. Transfection of siRNA was performed after the release from the first thymidine block. Attached cells were harvested at 8 h after releasing from the second thymidine block (G_2_ phase). Nocodazole was added at 8 h after the cells were released from the second thymidine block. After another 6 h, mitotic cells were harvested by mechanical shake off.

### RNA interference

Cells were transfected with siRNA (10 nM unless stated otherwise) using Lipofectamine^TM^ RNAiMAX (Life Technologies). Stealth siRNA targeting AURKA (GGCCAAUGCUCAGAGAAGUACUUGA), AURKB (UCUUAGGGCUCAAGGGAGAGCUGAA), and PLK1 (CAGCCUGCAGUACAUAGAGCGUGAU) were obtained from Life Technologies.

### Flow cytometry

Flow cytometry analysis after propidium iodide staining was performed as described previously [46].

### Live-cell imaging

Cells were seeded onto 24-well culture plates and imaged using a Ti-E inverted fluorescent microscope (Nikon, Tokyo, Japan) equipped with a SPOT BOOST EMCCD camera (Diagnostic Instrument, Sterling Heights, MI, USA) and an INU-NI-F1 temperature, humidity, and CO_2_ control system (Tokai Hit, Shizuoka, Japan).

### Infrared imaging

Infrared images of cells expressing iRFP were acquired and quantified with an Odyssey CLx system (LI-COR Biosciences, Lincoln, NE, USA).

### Antibodies and immunological methods

Antibodies against CDK1 [47] and cyclin B1 [41] were obtained from sources as described previously. Antibodies against β-actin (Sigma-Aldrich), AURKA, CDC27, cleaved PARP1(Asp214), and phospho-PLK1^Thr210^ (BD Biosciences, Franklin Lakes, NJ, USA), AURKB (Sigma-Aldrich), phospho-AURKA^Thr288^/AURKB^Thr232^/AURKC^Thr198^ (Cell Signaling Technology, Beverly, MA, USA), cleaved caspase 3 (using a Apoptosis Western Blot Cocktail, Abcam, Cambridge, UK), phospho-histone H3^Ser10^ and PLK1 (Santa Cruz Biotechnology, Santa Cruz, CA, USA) were obtained from the indicated suppliers. Immunoblotting was performed as described [44].

### Tumor xenografts

The experimental protocol was evaluated and approved by the Animal Care Committee, HKUST. HONE1 cells (2 × 10^7^) were injected subcutaneously into both sides of the dorsa of 4-6-week-old female BALB/c athymic (nude) mice. Three animals per group were used in each experiment. Tumors were regularly measured using a Vernier caliper. Volume was calculated according to the formula: π/6×length×width^2^ [48]. Day = 0 was designated as when the tumor volume was ~100 mm^3^. BI 2536 (20 mg/kg) was administrated with intraperitoneal injection into the mice daily from day 1 to 5. Mice were killed when tumors reached 1,000 mm^3^.

## SUPPLEMENTAL FIGURES AND VIDEOS


