# Supplementary material for: Co-inhibition of polo-like kinase 1 and Aurora kinases promotes mitotic catastrophe
Source: Oncotarget. 2015 Mar 20;6(11):9327–40. doi: 10.18632/oncotarget.3313 (PMC4496220; doi:10.18632/oncotarget.3313)
Supplement: Supplementary file 1 [file oncotarget-06-9327-s001.pdf]

SUPPLEMENTAL FIGURES AND VIDEOS

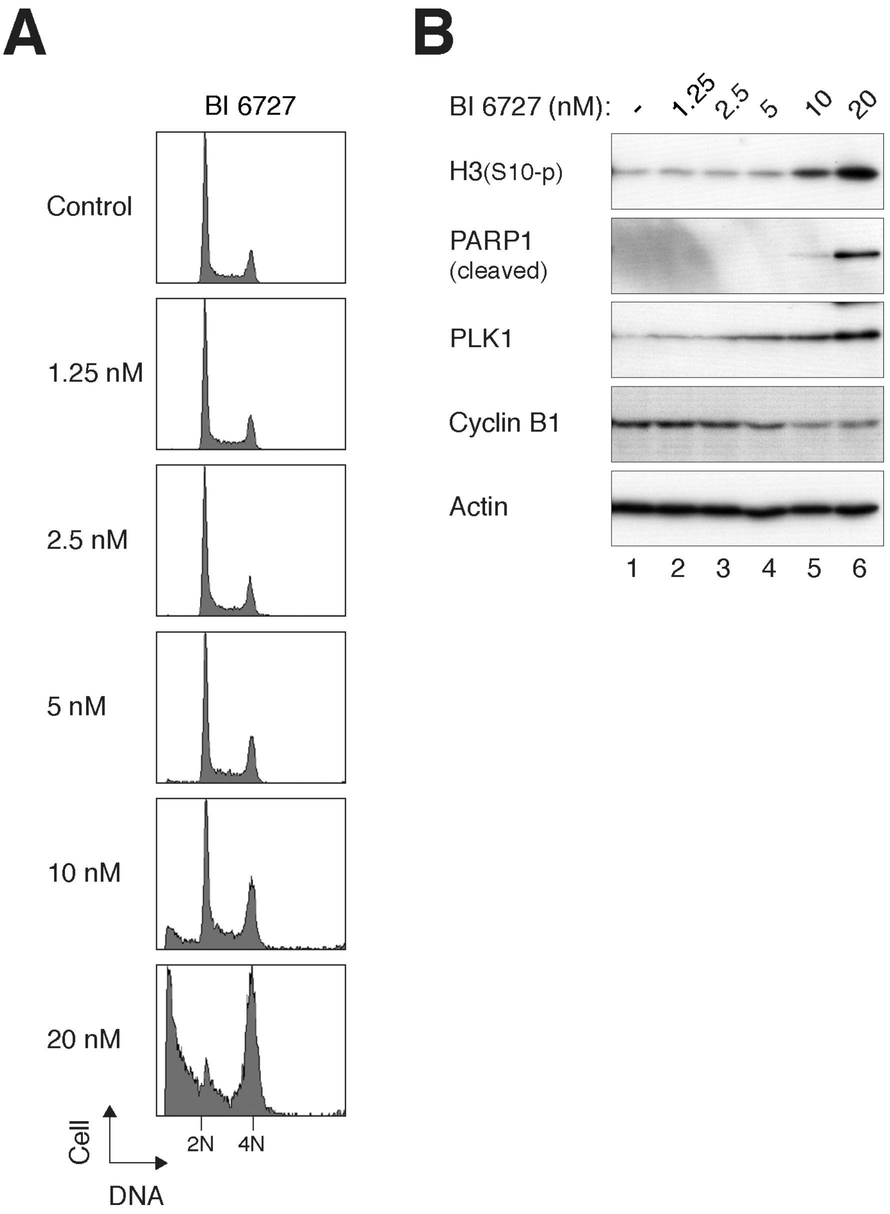

Supplementary Figure S1: Inhibition of PLK1 with BI 6727 induces mitotic catastrophe. (A) BI 6727 promotes G_2/M delay and apoptosis in a dose-dependent manner. HeLa cells were incubated with buffer or the indicated concentrations of BI 6727. After 24 h, the cells were harvested and the DNA content was analyzed with flow cytometry. The positions of 2N and 4N DNA content are indicated. **(B)** BI 6727 induces mitotic catastrophe. HeLa cells were incubated with buffer or the indicated concentrations of BI 6727 for 24 h. Lysates were prepared and the indicated proteins were detected with immunoblotting. Actin analysis was included to assess protein loading and transfer.

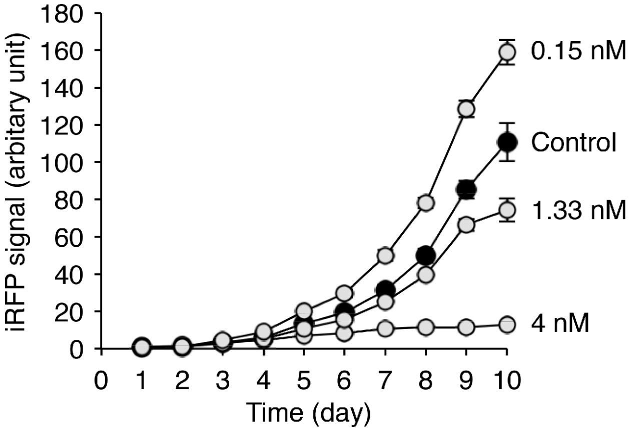

Supplementary Figure S2: Low concentrations of PLK1i stimulate cell proliferation in HONE1. HONE1 cells expressing iRFP (~200 cells) were seeded onto 6-well culture plates and cultured in the presence of buffer or different concentrations of PLK1i. On different days, the plate was scanned with an Odyssey infrared imaging system and the iRFP signal was quantified (average ±SD of three independent experiments). Note that the PLK1i was left in the medium continuously throughout the experiment.

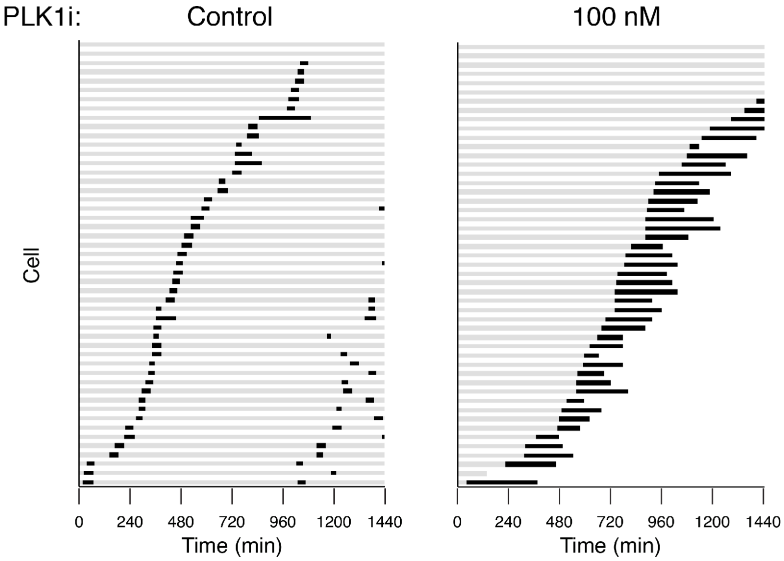

Supplementary Figure S3: High concentrations of PLK1i induce delay in early mitosis. HeLa cells expressing histone H2B-GFP were exposed to buffer or 100 nM of PLK1i. Individual cells were then tracked for 24 h with time-lapse microscopy. Each horizontal bar represents one cell (n = 50). Light grey: interphase; black: mitosis (from DNA condensation to anaphase or mitotic slippage); truncated bars: cell death.

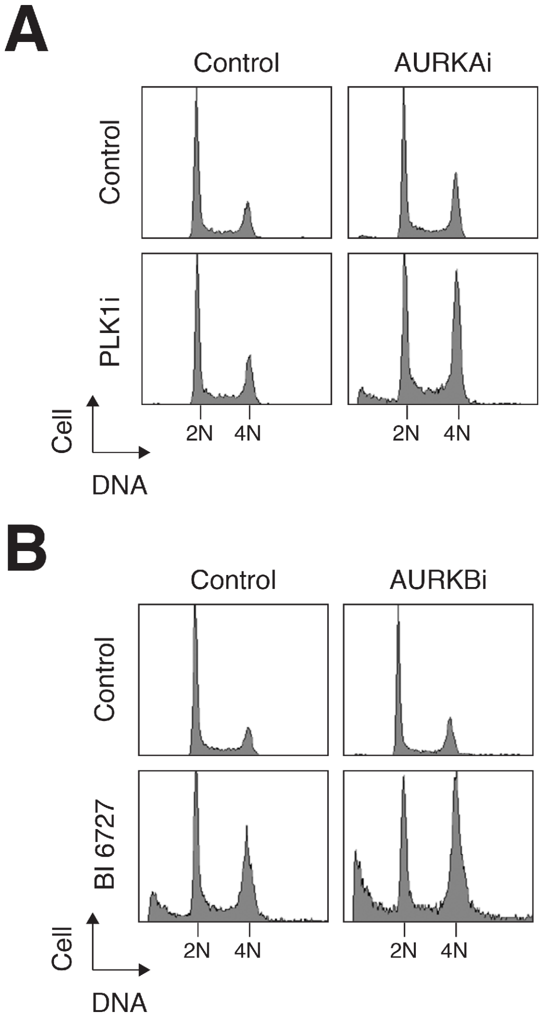

Supplementary Figure S4: BI 6727 cooperates with Aurora kinase inhibitors to induce mitotic catastrophe. **(A)** BI 6727 cooperates with AURKAi to induce G_2/M delay. HeLa cells were incubated with BI 6727 (5 nM) and/or AURKAi (250 nM) as indicated. After 24 h, the cells were harvested and analyzed with flow cytometry. The positions of 2N and 4N DNA content are indicated. **(B)** BI 6727 cooperates with AURKBi to induce G_2/M delay. HeLa cells were incubated with BI 6727 (10 nM) and/or AURKBi (25 nM) as indicated. After 24 h, the cells were harvested and analyzed with flow cytometry.

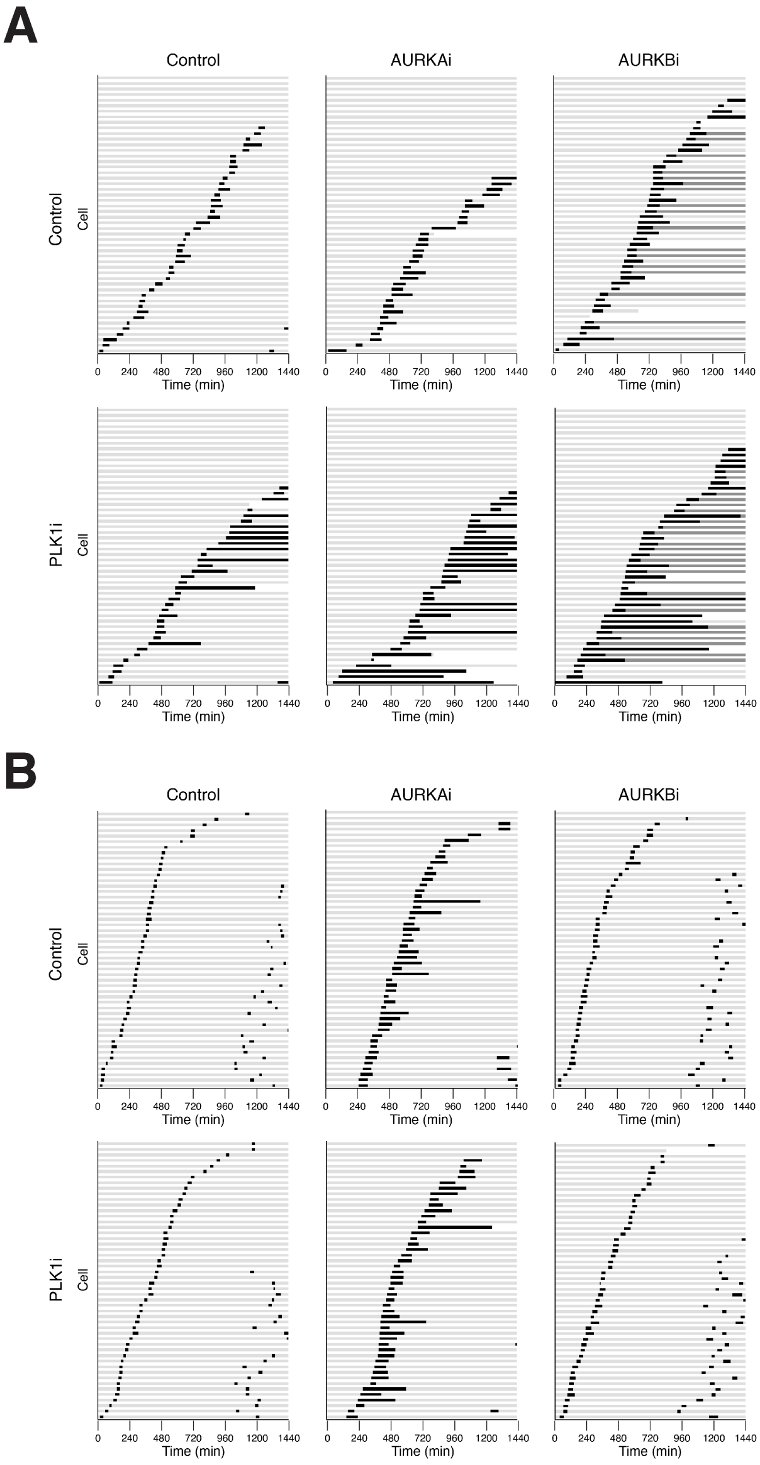

Supplementary Figure S5: Co-inhibition of PLK1 and Aurora kinases specifically sensitizes NPC cells. **(A)** PLK1i cooperates with Aurora kinase inhibitors to induce mitotic arrest and slippage. HONE1 cells expressing histone H2B-mRFP were incubated with PLK1i (2.5 nM), AURKAi (250 nM), and AURKBi (25 nM). Individual cells were then tracked for 24 h with time-lapse microscopy. Each horizontal bar represents one cell (n = 50). Light grey: interphase; black: mitosis (from DNA condensation to anaphase or mitotic slippage); dark grey: mitotic slippage; truncated bars: cell death. **(B)** NP460 cells are not affected by combinatorial treatment with PLK1i, AURKAi, and AURKBi. NP460 cells expressing histone H2B-GFP were incubated with a combination of PLK1i (2.5 nM), AURKAi (250 nM), and AURKBi (25 nM). Individual cells were then tracked for 24 h with time-lapse microscopy. Each horizontal bar represents one cell (n = 50). Light grey: interphase; black: mitosis (from DNA condensation to anaphase or mitotic slippage); truncated bars: cell death. Only one of the daughter cells after cell division was tracked.

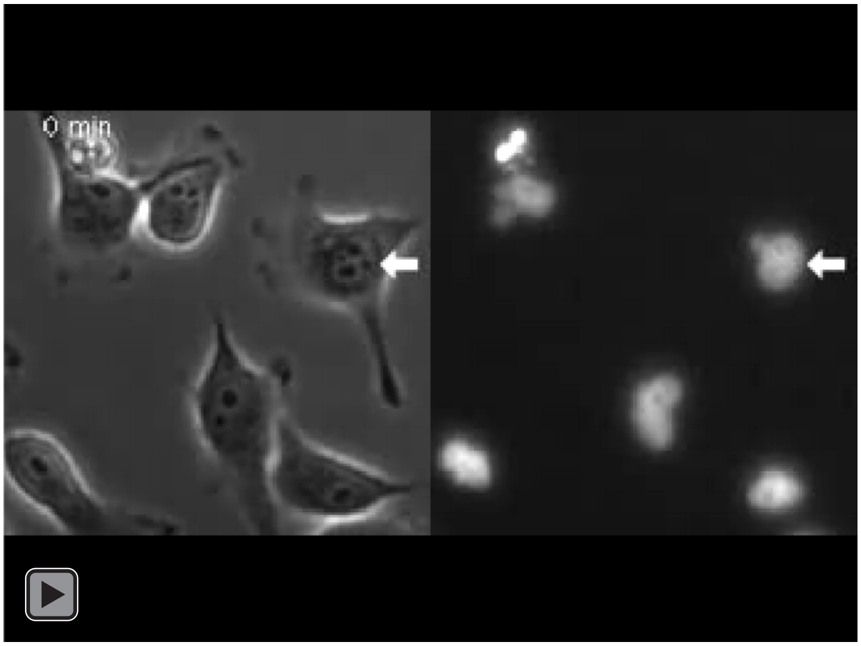

Supplementary Video 1: Live-cell imaging of control HeLa cells. HeLa cells expressing histone H2B-GFP were monitored with time-lapse microscopy (5 min/frame). Channels for bright field (left) and histone H2B-GFP (right) are shown.

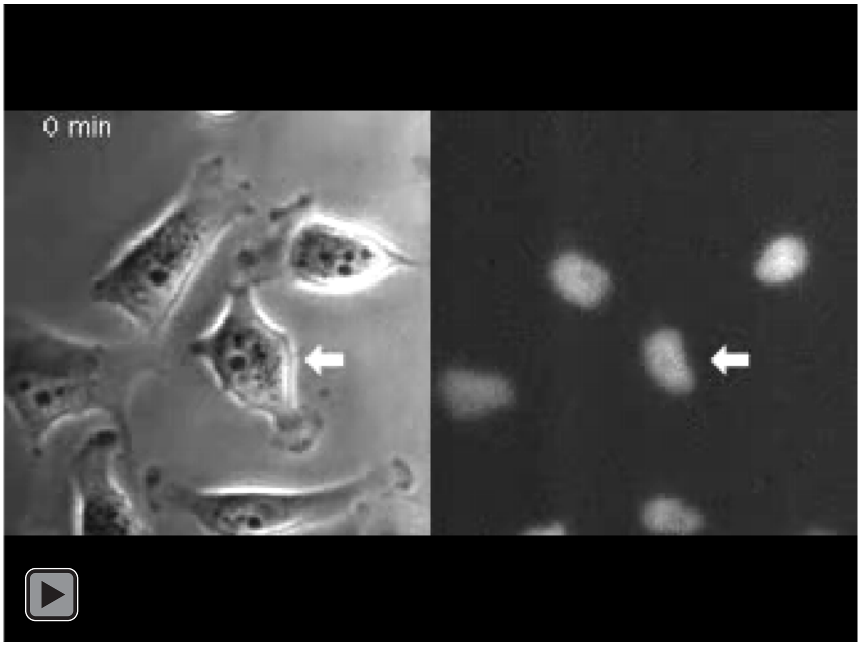

Supplementary Video 2: Inhibition of PLK1 induces metaphase arrest. HeLa cells expressing histone H2B-GFP were incubated with 2.5 nM of PLK1i and monitored with time-lapse microscopy (5 min/frame). Channels for bright field (left) and histone H2B-GFP (right) are shown. The highlighted cell was trapped in metaphase and was unable to undergo anaphase. The metaphase plate flapped on its axis (with the appearance of losing chromosomal alignment) before undergoing apoptosis.

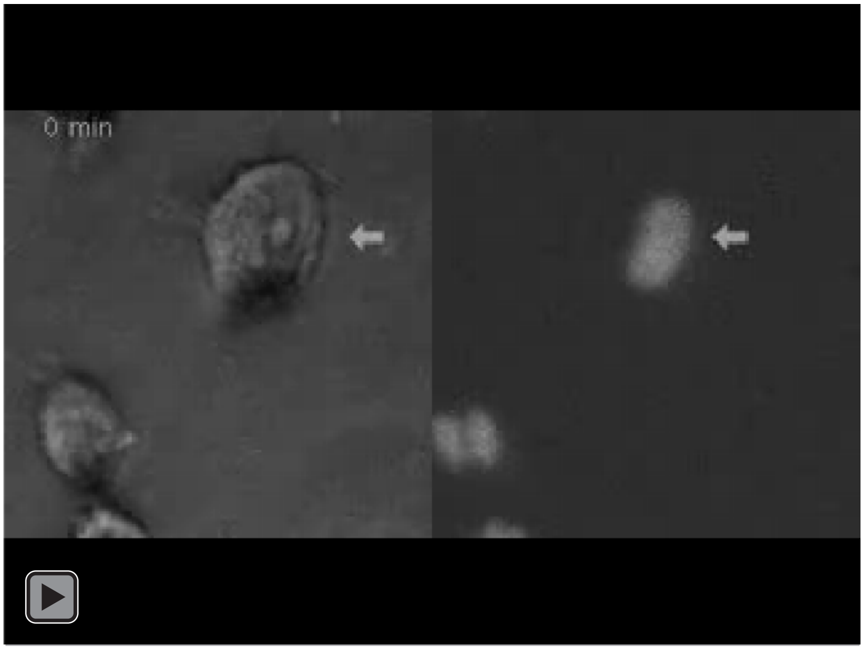

Supplementary Video 3: High concentrations of PLK1i induce an early mitotic arrest. HeLa cells expressing histone H2B-GFP were incubated with 100 nM of PLK1i and monitored with time-lapse microscopy (5 min/frame). Channels for bright field (left) and histone H2B-GFP (right) are shown.

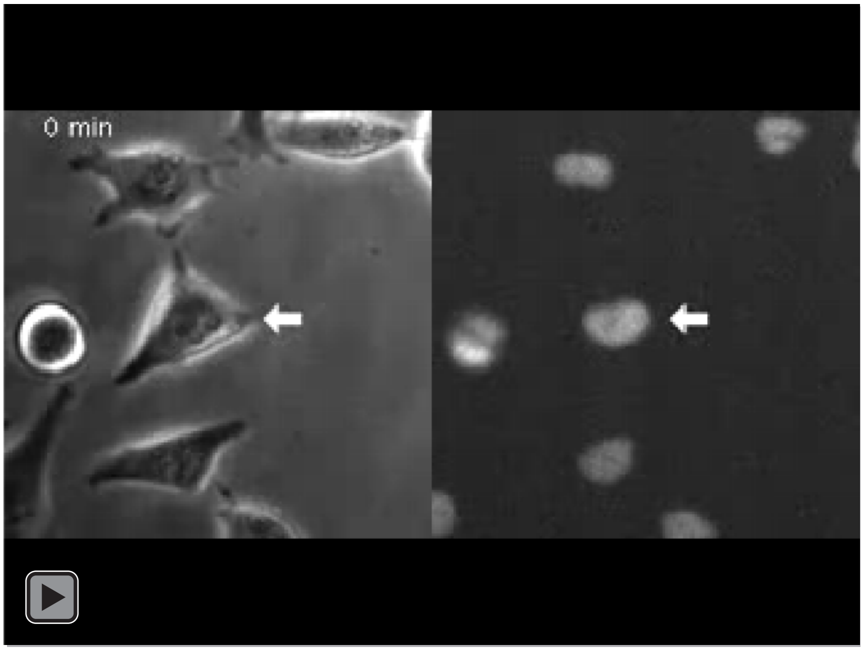

Supplementary Video 4: Inhibition of AURKA induces a delay in mitosis after the metaphase plate is formed. HeLa cells expressing histone H2B-GFP were incubated with 1 µM of AURKAi and monitored with time-lapse microscopy (5 min/frame). Channels for bright field (left) and histone H2B-GFP (right) are shown. A representative cell that displayed a delay in mitosis after the metaphase was formed is highlighted.

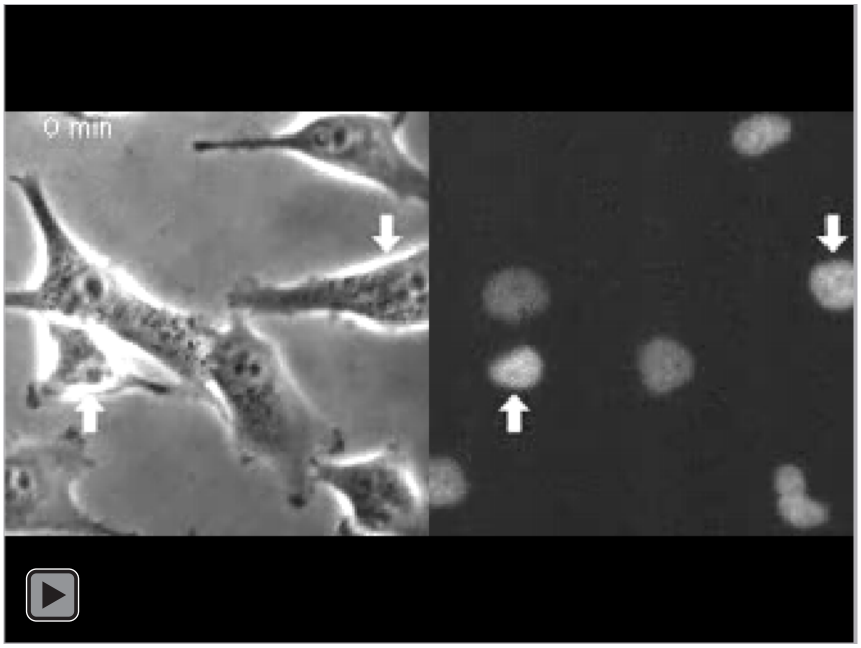

Supplementary Video 5: Inhibition of AURKA and PLK1 together induces mitotic block and cell death. HeLa cells expressing histone H2B-GFP were incubated with AURKAi (250 nM) and PLK1i (2.5 nM) and monitored with time-lapse microscopy (5 min/frame). Channels for bright field (left) and histone H2B-GFP (right) are shown. Two representative cells that were trapped in mitosis before undergoing apoptosis are highlighted.

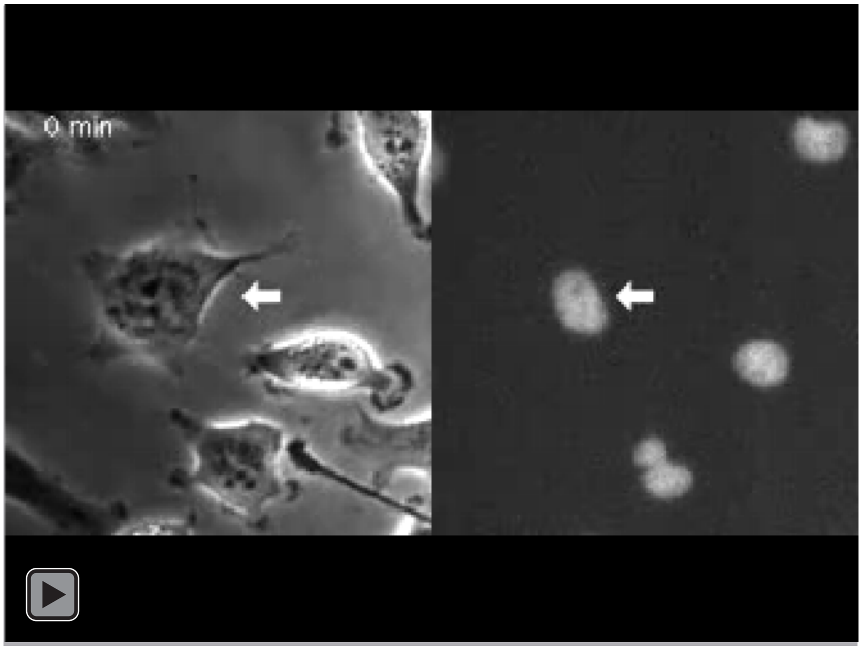

Supplementary Video 6: Inhibition of AURKB induces mitotic slippage. HeLa cells expressing histone H2B-GFP were incubated with 50 nM of AURKBi and monitored with time-lapse microscopy (5 min/frame). Channels for bright field (left) and histone H2B-GFP (right) are shown.

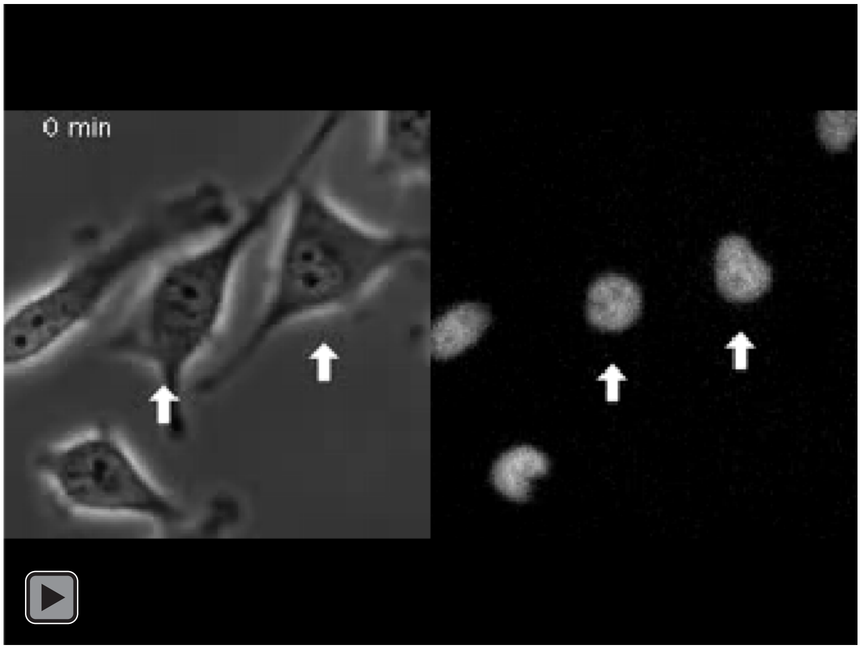

Supplementary Video 7: Inhibition of AURKB and PLK1 together induces mitotic slippage. HeLa cells expressing histone H2B-GFP were incubated with AURKBi (12.5 nM) and PLK1i (2.5 nM) and monitored with time-lapse microscopy (5 min/frame). Channels for bright field (left) and histone H2B-GFP (right) are shown. One of the highlighted cells (left) displayed extensive delay in mitosis before undergoing cell division. Mitotic slippage occurred in the other highlighted cell.
